# Person-to-Person Transmission During a Norovirus Outbreak in a Korean Kindergarten: A Retrospective Cohort Study

**DOI:** 10.3390/children12081027

**Published:** 2025-08-04

**Authors:** Yongho Park, Hyelim Jang, Jieun Jang, Ji-Hyuk Park

**Affiliations:** 1 Infectious Disease Control Division, Uijeongbu-si Public Health Center, Uijeongbu 11649, Republic of Korea; werqwer@korea.kr (Y.P.); hyelim90@korea.kr (H.J.); 2 Department of Preventive Medicine, Dongguk University College of Medicine, Gyeongju 38066, Republic of Korea; jieunjang@dongguk.ac.kr

**Keywords:** norovirus, outbreaks, person-to-person transmission, kindergartens, epidemiology

## Abstract

**Highlights:**

A norovirus outbreak occurred at a kindergarten with 28 cases out of 114 children (24.6%). Sharing the same class or floor as the primary case was a significant risk factor in this retrospective cohort study.

Isolation and restriction of symptomatic children should be strengthened, especially in kindergartens.

**What are the main findings?**

- This study confirmed person-to-person transmission as the primary route in a kindergarten norovirus outbreak.
- The person-to-person transmission was identified through statistical analysis.

**What is the implication of the main finding?**

- These findings suggest the need to systematically investigate norovirus outbreaks considering foodborne and person-to-person transmission.

**Abstract:**

**Objectives:** Norovirus outbreaks occur in densely populated environments, such as long-term care facilities, hospitals, and schools. On 22 October 2022, an outbreak of acute gastroenteritis was reported at a kindergarten in Korea. An epidemiologic investigation was conducted to identify the source of the infection and prevent further spread. **Methods**: Rectal swab and environmental samples were collected for bacterial and viral testing. A retrospective cohort study was conducted among 114 kindergarteners at the kindergarten. Relative risks (RRs) and 95% confidence intervals (CIs) were calculated to assess associations of contact with the primary case, as well as food and water consumption. **Results:** Of the kindergarteners, 28 out of 114 (24.6%) met the case definition. The primary case occurred on 19 October, and subsequent cases began on 21 October. Sharing the same four-year-old class as the primary case (RR, 2.56; 95% CI, 1.35–4.87), being in the same regular class (RR, 2.37; 95% CI, 1.27–4.41), being on the same floor during after-school class (RR, 3.49; 95% CI, 1.74–7.00), and attending the same English class (RR, 1.98; 95% CI, 1.05–3.72) were statistically significant. Consumption of drinking water on the third floor and fourth floor on 20 October had significantly higher and lower RRs, respectively. Norovirus was detected in 9 out of 18 rectal swab samples (50.0%). **Conclusions:** This norovirus outbreak at the kindergarten was presumed to have been caused by person-to-person transmission from the primary case. Isolation and restriction of symptomatic children in kindergartens should be thoroughly implemented. Additionally, enhanced surveillance among family members of affected individuals is necessary to prevent further outbreaks.

## 1. Introduction

Norovirus belongs to the Caliciviridae family and is classified into genogroups GI–GVII. Among these, the GI, GII, and GIV genogroups infect humans and can cause acute gastroenteritis [1]. Human norovirus infections account for approximately 18% of global cases and are the leading cause of acute gastroenteritis [2]. Norovirus can be transmitted not only through the consumption of contaminated food or water, but also through contact with an infected person or contaminated surfaces and objects [3]. In the United States, over 60% of norovirus outbreaks occur in densely populated environments, such as long-term care facilities, hospitals, and schools, with person-to-person transmission being the primary route [3,4]. Norovirus is efficiently transmitted from person to person due to its low infectious dose, high viral loads in vomitus and feces, and relatively long excretion period in feces up to 8 weeks [5,6].

The incubation period for norovirus infection ranges from 12 to 48 h [7]. After this period, symptoms such as vomiting, diarrhea, nausea, abdominal cramps, and mild fever may develop. In healthy individuals, these symptoms typically resolve within 48 h. However, children and the elderly are at a higher risk of developing more severe and prolonged symptoms that may lead to hospitalization [8]. Norovirus shedding can continue even after symptoms have resolved [5]. Therefore, contact with norovirus patients should be avoided for at least 48 h after symptom resolution [9]. Individuals who have been in contact with an infected person should be monitored for three days [7].

In Korea, outbreak investigations are initiated when two or more individuals develop similar symptoms such as diarrhea or vomiting after consuming the same food (including drinking water) [10]. In 2022, a total of 492 foodborne disease outbreaks were reported nationwide. The most common pathogen was norovirus (31.9%), followed by *Salmonella* spp. (8.1%), pathogenic *Escherichia coli* (6.5%), and *Campylobacter* spp. (3.7%) [11]. From 2019 to 2023, data from the Enteric Pathogen Active Surveillance Network (Enter-Net) in Korea indicated that norovirus was the most frequently detected viral pathogen (69.2%) among patients with gastroenteritis. Other detected viruses included astrovirus (9.5%), group A rotavirus (9.2%), and intestinal adenovirus (7.8%) [12].

On 22 October 2022, a kindergartener’s parent reported an outbreak to the Gyeonggido Office of Education. The kindergarten subsequently reported it to the Uijeongbu City Health Center. The Uijeongbu City Health Center conducted an outbreak investigation to identify the source of infection and implement control measures to prevent further transmission.

## 2. Materials and Methods

### 2.1. Outbreak Setting and Case Definition

**Figure A1:**
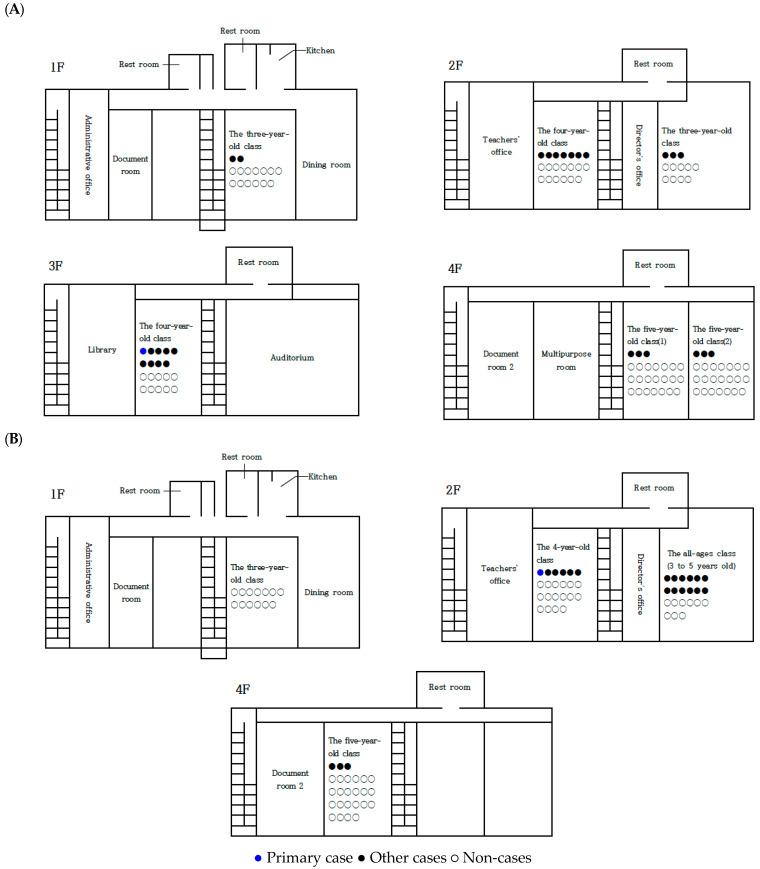
Floor plan of a kindergarten and case distribution in the morning (**A**) and afternoon (**B**).

On the afternoon of 22 October, the kindergarten reported an outbreak involving approximately 20 kindergarteners with symptoms such as vomiting, fever, and diarrhea. The kindergarten consisted of six regular classes in the morning, with two classes for each age group from 3 to 5 years old. The morning three-year-old classes were located on the first and second floors, the morning four-year-old classes on the second and third floors, and the morning five-year-old classes (A and B) on the fourth floor. After the morning regular classes, kindergarteners ate lunch in order from the youngest in the dining room on the first floor (Figure A1A).

After lunch, some kindergarteners participated in after-school classes in the afternoon, while others returned home. The kindergarten had four after-school classes: the afternoon three-year-old class on the first floor, the afternoon four-year-old class on the second floor, the afternoon five-year-old class on the fourth floor, and an all-ages afternoon class on the second floor (Figure A1B). The afternoon four-year-old class and the all-ages afternoon class were on the same floor, allowing participants to move between the two classes on the second floor.

Some kindergarteners who participated in the after-school classes also attended English classes on Wednesdays (including 19 October). Four-year-old and five-year-old kindergarteners took English classes on the second floor (afternoon four-year-old class) and the fourth floor (next to the afternoon five-year-old class), respectively (Figure A1B). Cases were defined as kindergarteners from the kindergarten who experienced vomiting or diarrhea (defined as ≥3 loose stools within any 24 h period) between 19 October and 23 October.

### 2.2. Epidemiological Investigation and Site Inspection

A retrospective cohort study was conducted on all kindergarteners at the kindergarten to identify risk factors associated with the illness. We modified standard questionnaires from the Korea Disease Control and Prevention Agency to collect information on demographic characteristics, clinical symptoms, and food and water consumption at the kindergarten [13]. Since the kindergarteners were too young to describe clinical symptoms and food and water intake, the information was collected by their parents and homeroom teachers, respectively. Meals provided at the kindergarten included morning snacks, lunch, and afternoon snacks. The kindergarten was affiliated with an elementary school, and lunch meals were prepared in the kitchen of the elementary school.

We visited the dining room of the kindergarten and the kitchen of the elementary school to assess hygienic conditions. Interviews with the director and homeroom teachers were conducted to obtain information on the general characteristics of the kindergarten. Additionally, information on participation in regular classes, after-school classes, and English classes was collected through the staff at the kindergarten. Interviews were conducted by trained public health officials.

### 2.3. Sample Collection, Isolation, and Detection of Pathogens

Rectal swab samples were recommended for symptomatic kindergarteners at the kindergarten and all food handlers at the elementary school. Rectal swabs were obtained instead of fecal samples for rapid specimen collection during field investigations. On 23 October, we visited the kitchen of the elementary school and collected preserved food samples served between 19 and 21 October, which had been stored below minus 18 °C.

From the kindergarten, forty-one environmental samples including door hooks, desks, and toilet handles were collected. From the elementary school, we collected seven samples from cooking utensils and one sample from cooking water. From the kindergarten, we collected one sample from drinking water in the dining room and three samples from water purifiers. Preserved food, cooking utensils, and environmental samples were tested for ten bacterial species known to cause gastrointestinal infections. A total of forty-one environmental samples and three types of kimchi (non-heated foods) were additionally tested for norovirus. Cooking water samples were analyzed for general bacteria including *E. coli*. Samples from drinking water and water purifiers were tested for *E. coli* and *Salmonella* spp. Laboratory analyses were conducted at the Northern Branch of the Gyeonggi Province Institute of Health and Environment. The selection of tested pathogens followed the Waterborne and Foodborne Infectious Disease Guideline issued by the Korea Disease Control and Prevention Agency (KDCA), which recommends screening for 10 bacterial pathogens and 5 viral pathogens commonly implicated in outbreaks: *Shigella* spp., pathogenic *E*. *coli*, *Salmonella* spp., *Vibrio parahaemolyticus*, *Campylobacter jejuni*, *Clostridium perfringens*, *Staphylococcus aureus*, *Bacillus cereus*, *Yersinia enterocolitica*, and *Listeria monocytogenes* for bacteria and Group A rotavirus, astrovirus, enteric adenovirus, norovirus, and sapovirus for virus.

### 2.4. Genotypic Analysis

Genotyping was performed on norovirus detected in the rectal samples. Norovirus was identified using real-time reverse transcription polymerase chain reaction (RT-PCR; PowerChek™ Norovirus GI/GII Multiplex Real-time PCR kit, Kogene Biotech Corp., Seoul, Republic of Korea). For viral genotyping, a conventional RT-PCR (HyQTM One step-RT-PCR PreMix kit, SNC Corp., Gyeonggi-do, Republic of Korea) was performed using norovirus-specific primers. PCR products were confirmed by capillary electrophoresis and sent to a sequencing service provider (Macrogen, Seoul, Republic of Korea) for nucleotide analysis. The genotypes were identified using Basic Local Alignment Search Tool provided by the National Center for Biotechnology Information.

### 2.5. Statistical Analysis

Statistical analyses were performed using Epi Info Version 7.2.5.0 (CDC, Atlanta, GA, USA). Relative risks (RRs) and 95% confidence intervals (CIs) were calculated to identify risk factors associated with the illness. Fisher’s exact test was applied to compare attack rates and statistical significance was set at *p* < 0.05.

## 3. Results

### 3.1. General Characteristics of the Kindergarten Cases

Out of the 114 kindergarteners at the kindergarten, 28 cases (attack rate: 24.6%) were identified. The most common symptom was vomiting (96.4%), followed by abdominal pain (67.9%), fever (57.1%), and diarrhea (21.4%). In the 3-year-old group (*n* = 6), five kindergarteners (83.3%) experienced vomiting and one (16.7%) had diarrhea. In the 4-year-old group (*n* = 16), vomiting was reported in 16 kindergarteners (100%) and diarrhea in 5 (31.3%). In the 5-year-old group (*n* = 6), six (100%) experienced vomiting and one (16.7%) had diarrhea. Out of 28 cases, 15 cases (53.6%) were boys. The majority occurred in the four-year-old group (16 cases, 57.1%). Among the regular classes, the cases in the morning four-year-old class on the third floor were the highest (9 cases, 32.1%), followed by the morning four-year-old class on the second floor (7 cases, 25.0%) and the morning three-year-old class on the second floor (4 cases, 14.3%). Among the after-school classes, the cases in the afternoon all-ages class on the second floor were the highest (13 cases, 46.4%), followed by the afternoon four-year-old class on the second floor (6 cases, 21.4%) and those who did not participate in the after-school classes (6 cases, 21.4%). Among the English classes on October 19, the most common participants were the four-year-old English class on the second floor (10 cases, 35.7%), although the majority of cases did not participate in the English classes (12 cases, 42.9%; Table 1).

### 3.2. Primary Case and Epidemic Curve

**Figure 1:**
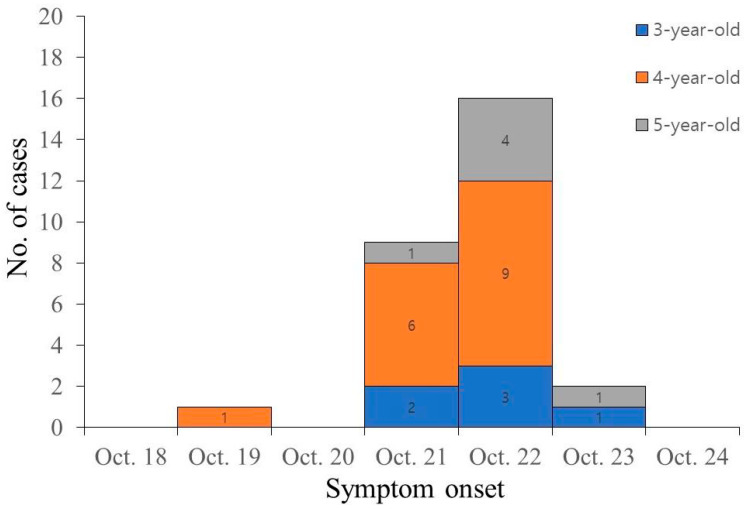
Epidemic curve of the norovirus outbreak at a kindergarten in 2022. The cases of three- and five-year-olds on October 21 participated in the afternoon all-ages class on the second floor.

**Figure A2:**
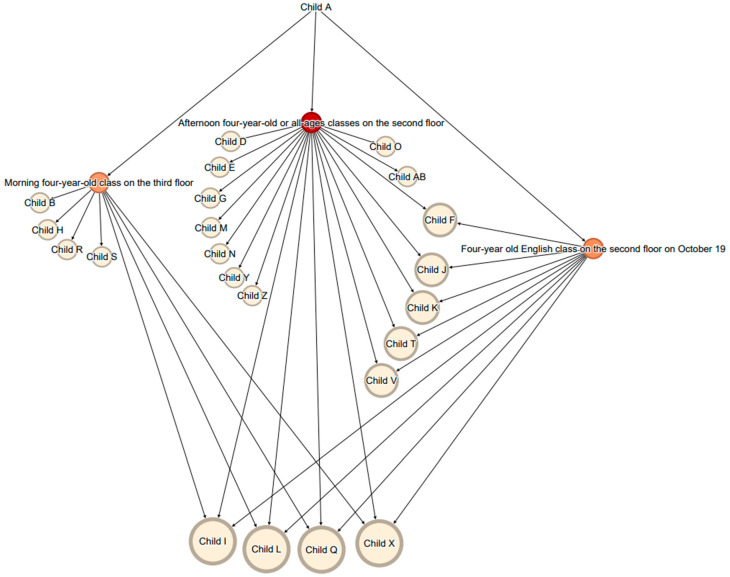
Cases summary and presumed risk exposure locations in a kindergarten.

The primary case was a four-year-old boy who belonged to the morning four-year-old class on the third floor. The kindergartener experienced diarrhea around 10 a.m. on 19 October and vomited eight times at the kindergarten. However, he remained at the kindergarten that day and continued attending classes from 19 to 21 October. The primary case participated in the afternoon four-year-old class on the second floor and the four-year-old English class on the second floor on 19 October. There were no additional cases on 20 October. Cases began to occur again on 21 October, with a peak of 16 cases on 22 October (Figure 1). Of the nine cases that occurred on 21 October, six were four-year-olds. The other three cases (two three-year-olds and one five-year-old) participated in the afternoon all-ages class on the second floor (Figure A2). Despite multiple vomiting episodes, the primary case was not isolated.

### 3.3. Association of Food and Water Consumption at the Kindergarten

The kindergarten received the same lunch from the elementary school. The lunch was prepared in the kitchen of the elementary school, brought to the kindergarten, and served in the dining room. Out of 250 students at the elementary school, no cases were reported through symptom surveillance. The kindergarten served morning and afternoon snacks provided as prepackaged products by a separate vendor. This vendor also supplied 13 other kindergartens, and no additional cases were reported. All cases, except the primary case, occurred between 21 and 23 October. Food items served on 20 October were not significantly associated with the illness. The RRs of water from the purifiers on the third floor (2.15; 95% CI, 1.10–4.17) and the fourth floor (0.40; 95% CI, 0.16–0.97) on 20 October were significantly higher and lower, respectively (Table 2).

### 3.4. Association of Contact with the Primary Case at the Kindergarten

The four-year-old group had a significantly higher RR (2.56; 95% CI, 1.35–4.87). Male sex was not significantly associated with the illness. The primary case could visit the afternoon all-ages class on the same floor. The RR for the morning four-year-old class on the third floor (2.37; 95% CI, 1.27–4.41) was significantly higher than for other morning classes. The afternoon four-year-old class on the second floor was not significantly associated with the illness. The afternoon all-ages class on the second floor and combined afternoon four-year-old or all-ages classes on the second floor had significantly elevated RRs of 3.84 (95% CI, 2.17–6.80) and 3.49 (95% CI, 1.74–7.00), respectively. The primary case participated in the four-year-old English class on the second floor on 19 October, and the RR (1.98; 95% CI, 1.05–3.72) was significantly higher (Table 3).

### 3.5. Laboratory Findings

**Figure A3:**
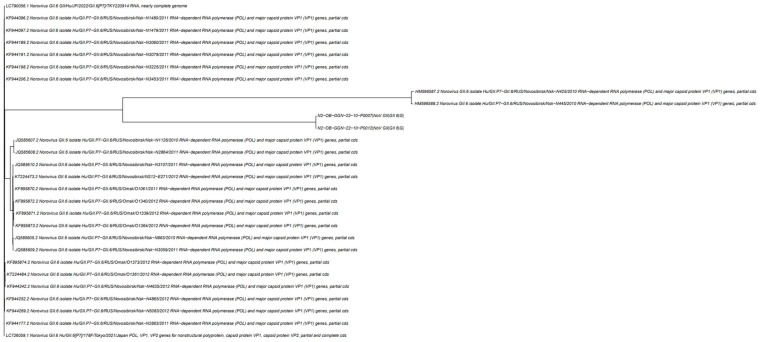
Genetic relationships between norovirus GII.6 strains of this outbreak and reference sequences (bootstrap ≥50%).

Out of 18 rectal swab samples collected from the kindergarten cases, norovirus was detected in 9 samples (50.0%). Norovirus GII.6 was identified in two samples, including that of the primary case. Phylogenetic analysis including Korean, neighboring, and distant GII.6 strains showed that the two Korean sequences clustered together and were most closely related to the Russian strains (Figure A3). The remaining seven noroviruses were identified as GII only; subtyping could not be performed due to insufficient sample volumes. Rectal swab and forty-one environmental samples collected from the kindergarten were negative for bacteria. Six rectal swab samples collected from food handlers at the elementary school were negative for bacteria and viruses. Samples from preserved foods and cooking utensils were negative for bacteria. Cooking water samples were negative for general bacteria, including *E. coli*. Samples from drinking water and water purifiers were negative for *E. coli* and *Salmonella* spp. Norovirus test were negative for samples from kimchi and the environment.

## 4. Discussion

In this norovirus outbreak, the primary case occurred on 19 October, and subsequent cases followed on 21 October. The characteristics of the primary case, including the four-year-old group, the morning four-year-old class on the third floor, the afternoon four-year-old or all-ages classes on the second floor, and the four-year-old English class, were significantly associated with the illness. Consumption of purified water on the third floor on 20 October had a significantly higher RR, while that on the fourth floor had a significantly lower RR. Restriction of symptomatic cases or cases within the kindergarten was recommended. Furthermore, environmental disinfection and health education were conducted on 23 October. No additional cases were reported after 24 October.

Kindergarteners are too young to maintain strict personal hygiene practices such as hand washing [14]. The same characteristics as the primary case were significantly associated with illness. The primary case was not isolated and vomited eight times at the kindergarten. Norovirus could be transmitted through exposure to aerosolized particles from vomitus as well as direct contact with vomitus [15]. Thus, this norovirus outbreak was likely caused by person-to-person transmission from the primary case.

One of the main routes of norovirus transmission is the ingestion of contaminated food or water [16,17]. In Korea, norovirus outbreaks caused by contaminated cabbage kimchi and seafood have been reported [18,19]. Food items served at the kindergarten on 20 October were not significantly associated with illness. Additionally, the same lunch was served at the elementary school, and no cases were reported. Water from the purifiers on the third and fourth floors on 20 October had significantly higher and lower RRs, respectively. These associations might have been influenced by the primary case, who stayed on the third floor in the morning and on the second floor in the afternoon. Norovirus outbreaks caused by food or water consumption tend to exhibit a greater diversity of norovirus genotypes contrary to those caused by person-to-person transmission [20,21]. The norovirus genotypes identified in this outbreak were the same (GII.6).

The most common symptom in cases of the norovirus outbreak at the kindergarten was vomiting, which occurs more frequently than diarrhea in children infected with norovirus [22]. In settings with children, such as kindergartens and elementary schools, norovirus may spread more readily through exposure to vomitus [23]. Furthermore, childcare facilities are particularly vulnerable to the spread of norovirus due to prolonged indoor exposure [24]. Restriction and isolation in childcare facilities are effective in controlling norovirus outbreaks [14]. However, the cases were not restricted or isolated before the outbreak was reported.

Norovirus GII.4 has been the most predominant genotype since the mid-1990s [7]. In 2022, norovirus outbreaks in kindergartens and daycare centers increased rapidly in the metropolitan areas of Korea (Seoul, Gyeonggi-do, and Incheon), where the kindergarten is located [25]. According to data from the Enter-Net in Korea from 2019 to 2023, the predominant norovirus genotype among children under five years old was GII.4. In addition, GII.6 emerged as one of the major genotypes, with a nationwide detection rate exceeding 5% in both 2020 and 2021 [12]. In Japan, norovirus GII.6, along with GII.4, has also been reported as one of the main genotypes among young children [26]. As reports of GII.6 among young children are increasing in Japan, continuous monitoring is also needed in Korea.

We could not determine how the primary case was infected. Transmission through contact with asymptomatic norovirus carriers or ingestion of contaminated food might be possible, considering the proportion of asymptomatic infections ranges from 11% to 31% [5,27]. Norovirus could also spread among family members of cases and potentially be transmitted to other childcare facilities or schools [28]. However, household surveillance for norovirus infection was passive according to the guidelines in Korea and was not thoroughly conducted [10]. Additionally, the standard questionnaires for foodborne disease outbreaks in Korea did not include questions regarding person-to-person transmission [10]. Since norovirus is the most common cause of foodborne disease outbreaks in Korea [10], incorporating questions to assess person-to-person transmission such as contact with the primary case and household contacts into the questionnaire is necessary.

There are several limitations to this outbreak investigation. First, we could not obtain information about the exact vomiting sites of the primary case within the kindergarten. In addition, environmental samples could not be collected from the vomiting sites. Second, information about the cases was obtained by surrogates. Third, rectal swab samples could not be collected from all kindergarteners, which could lead to misclassification of cases. Fourth, active and broad household surveillance was not performed.

## 5. Conclusions

This report presents an outbreak of norovirus in Korea in which person-to-person transmission was identified through statistical analysis. These findings suggest the need to systematically investigate norovirus outbreaks considering foodborne and person-to-person transmission. The norovirus outbreak at the kindergarten was assumed to have been caused by person-to-person transmission from the primary case. The primary case was not restricted or isolated despite symptoms of diarrhea and vomiting. Proper preventive management, such as restriction, isolation, symptom surveillance, and personal hygiene, can reduce norovirus infection in childcare facilities. Furthermore, the family members of cases need to be monitored more strictly, and symptomatic children should be promptly isolated to prevent additional outbreaks.

## Figures and Tables

**Table 1:** General characteristics of cases at a kindergarten.

Characteristics (*n* = 28)	Cases (%)
Sex	
Boys	15 (53.6)
Girls	13 (46.4)
Age, yr	
3	6 (21.4)
4	16 (57.1)
5	6 (21.4)
Regular class	
Morning three-year-old class on the first floor	2 (7.1)
Morning three-year-old class on the second floor	4 (14.3)
Morning four-year-old class on the second floor	7 (25.0)
Morning four-year-old class on the third floor	9 (32.1)
Morning five-year-old class A on the fourth floor	3 (10.7)
Morning five-year-old class B on the fourth floor	3 (10.7)
After-school class	
Afternoon three-year-old class on the first floor	0 (0.0)
Afternoon four-year-old class on the second floor	6 (21.4)
Afternoon five-year-old class on the fourth floor	3 (10.7)
Afternoon all-ages class on the second floor	13 (46.4)
Non-participants	6 (21.4)
English class on 19 October	
Four-year old English class on the second floor	10 (35.7)
Five-year old English class on the fourth floor	6 (21.4)
Non-participants	12 (42.9)

**Table 2:** Association between illness and food and water consumption at a kindergarten on 20 October.

Food and Water	Exposed			Unexposed			RR	95% CI	*p*-Value
	Cases	Total	AR (%)	Cases	Total	AR (%)			
Morning snack									
Milk	23	85	27.1	4	26	15.4	1.76	0.67–4.62	0.300
Lunch									
Sticky corn rice	27	105	25.7	1	9	11.1	2.31	0.35–15.11	0.449
Dried shrimp and Chinese cabbage soup	26	98	26.5	2	14	14.3	1.86	0.49–6.99	0.511
Soybean sprouts bulgogi	20	86	23.3	7	26	26.9	0.86	0.41–1.81	0.794
Stir-fried dried anchovies with green chili peppers	16	72	22.2	12	41	29.3	0.76	0.40–1.44	0.497
Cabbage kimchi	15	63	23.8	13	49	26.5	0.90	0.47–1.71	0.827
Mandarin	24	100	24.0	4	14	28.6	0.84	0.34–2.06	0.744
Afternoon snack									
Apple pancakes	21	72	29.2	7	42	16.7	1.75	0.81–3.76	0.177
High calcium milk	21	72	29.2	7	42	16.7	1.75	0.81–3.76	0.177
Water purifiers									
First floor	16	82	19.5	10	30	33.3	0.59	0.30–1.14	0.137
Second floor	7	17	41.2	20	96	20.8	1.98	0.99–3.94	0.118
Third floor	8	17	47.1	16	73	21.9	2.15	1.10–4.17	0.064
Fourth floor	5	41	12.2	22	72	30.6	0.40	0.16–0.97	0.038

AR, attack rate; RR, relative risk; CI, confidence interval. Fisher’s exact test was applied.

**Table 3:** Association between illness and contact with the primary case at a kindergarten.

Characteristics	Cases	Total	AR (%)	RR (95% CI)	*p*-Value
Sex					
Male	15	57	26.3	1.15 (0.61–2.20)	0.828
Female	13	57	22.8	Reference	
Age, yr					
4	16	39	41.0	2.56 (1.35–4.87)	0.005
Others	12	75	16.0	Reference	
Morning four-year-old class on the third floor					
Yes	9	19	47.4	2.37 (1.27–4.41)	0.018
No	19	95	20.0	Reference	
Afternoon four-year-old class on the second floor					
Yes	6	22	27.3	1.14 (0.53–2.47)	0.785
No	22	92	23.9	Reference	
Afternoon all-ages class on the second floor					
Yes	13	21	61.9	3.84 (2.17–6.80)	<0.001
No	15	93	16.1	Reference	
Afternoon four-year-old or all-ages classes on the second floor					
Yes	19	43	44.2	3.49 (1.74–7.00)	<0.001
No	9	71	12.7	Reference	
Four-year old English class on the second floor on October 19					
Yes	10	25	40.0	1.98 (1.05–3.72)	0.064
No	18	89	20.2	Reference	

AR, attack rate; RR, relative risk; CI, confidence interval. Fisher’s exact test was applied.

## Data Availability

Data available on request due to privacy.
